# Suicide Attempt by Dual Ethylene Glycol and Rat Poison Ingestion: Case Report

**DOI:** 10.1155/crps/5846563

**Published:** 2026-07-30

**Authors:** Danah Atassi, Amanda Bernardini, Wesley Wang, Christie Richardson

**Affiliations:** ^1^ Psychiatry Department, Inspira Health, Vineland, New Jersey, USA; ^2^ Psychiatry Department, Rowan-Virtua School of Osteopathic Medicine, Mount Laurel, New Jersey, USA

**Keywords:** case report, ethylene glycol, rat poison, suicide

## Abstract

Understanding the substances used in an overdose is informative to potential physiological complications and provides a guide for treatment modalities. This case describes a patient who overdosed on both ethylene glycol and rat poison, leading to a complex recovery. A 57‐year‐old female patient with a past psychiatric history of depression and alcohol use disorder presented to the emergency department unresponsive after a suicide attempt. The patient was found to have severe high‐anion‐gap metabolic acidosis, was intubated, and started on a bicarbonate drip and emergent hemodialysis. There was suspicion of toxic ingestion of ethylene glycol, given that lactate on arterial blood gas was significantly higher than serum lactic acid, so intravenous fomepizole was started. The patient’s family subsequently found a suicide note in the patient’s bedroom alongside a bottle of ethylene glycol and rat poison. Brain imaging revealed multiple lacunar infarcts in the bilateral cerebral hemispheres and diffusion abnormality in the hippocampi. Following medical stabilization and continued cognitive improvement over the course of hospitalization, the patient was discharged and referred to a partial hospitalization program for further psychiatric treatment. Reflecting on the patient’s unique hospital course, the overdose on both ethylene glycol and rat poison likely resulted in a physiological state similar to typical ethanol poisoning, with both acute and long‐term sequelae. Per standard treatment of care, fomepizole and a bicarbonate drip were started for treatment of both toxicities. From a psychiatric standpoint, the patient’s ongoing medical complications requiring hemodialysis resulted in difficulties with a direct discharge to an inpatient psychiatric facility following medical clearance; however, she was appropriate for a partial hospitalization level of care. This case highlights successful treatment that may be useful for those encountering similar patient case presentations.

## 1. Introduction

It is estimated that within developed countries, in a 1‐year period, ~0.3% of the population will attempt suicide [1]. Per data from the United States Poison Control, in 2023, 18.4% of exposures to poisons were intentional, and of the 27% of adult intentional ingestions, 19% were used for the suspected intent of a suicide attempt [2]. Understanding the substances used in overdoses is informative of the potential physiological complications that may ensue and provides a guide for treatment modalities. Additionally, determining the motives behind an overdose through a psychiatric evaluation can provide further guidance for appropriate treatment plans.

We present a case of a patient following the ingestion of both d‐CON rat poison and ethylene glycol in a suicide attempt. Other literature examples of d‐CON ingestion report substantial coagulopathy following ingestion of brodifacoum, including subarachnoid hemorrhage, with utilization of fresh frozen plasma and vitamin K1 for treatment [3–7]. These cases involved suspected greater quantities of rat poison ingested than the case being presented, and in one reviewed case, complications of ingestion led to the death of the patient [3]. Due to cholecalciferol within rat poison, ingestion can lead to increased levels of vitamin D, potentially progressing to toxicity [8, 9]. Vitamin D toxicity can subsequently cause hypercalcemia, which can impair kidney function [8, 9]. Cases detailing ethylene glycol ingestion describe physiological impacts on the central nervous, cardiac, pulmonary, and renal systems [10–12]. For ethylene glycol ingestion, management and prevention of further impairment can include the use of fomepizole as a means of alcohol dehydrogenase inhibition, and in critical cases, hemodialysis is used for treatment [10, 12–14].

To our knowledge, no other cases have been published in which this combination of toxic substances was ingested. Therefore, we present this case as an example of the physiological effects and psychosocial determinants of care following the ingestion of both rat poison and ethylene glycol. Additionally, we present this case to highlight successful treatment, which may benefit those encountering similar patient presentations (Supporting Information ).

## 2. Case Presentation

A 57‐year‐old female patient with a past medical history of hypertension and a past psychiatric history of depression and alcohol use disorder presented to the emergency department unresponsive with a Glasgow Coma Scale of 6. The family reported that the patient was slurring her speech on the phone, and during a check‐in a few hours later, they found her unresponsive with labored respirations. The patient was found to have severely elevated anion‐gap metabolic acidosis with an arterial blood gas showing a pH of 6.780, a bicarbonate level of 1.0, and a lactic acid level of 9.0. The patient’s calcium ranged from 7.4 to 9.1 mg/dL, with serum levels slowly normalizing. The patient was intubated, started on a bicarbonate drip, and transferred to the intensive care unit (ICU) for emergent hemodialysis. Table 1 shows a timeline of significant laboratory values and events.

**Table 1:** Significant events and findings during hospitalization.

Day	Significant events and findings
1	Admission to ICU after intubation, CRRT initiated, started on pressors Blood gases: pH 6.780; HCO_3_ 1 mmol/L; lactate 31 mmol/L Labs: lactic acid 9.0 mmol/L; ammonia 548 µmol/L; creatinine 1.26 mg/dL; ALT 14 units/L; AST 16 units/L; calcium 9.1 mg/dL CT abdomen and pelvis: mild right hydronephrosis with mild fullness in the right ureter
2	EEG: severe diffuse slowing Suicide note discovered by family next to bottles of ethylene glycol and rat poison (d‐CON) Fomepizole initiated Blood gases: pH 7.041; HCO_3_ 5.7 mmol/L; lactate 31 mmol/L Labs: lactic acid 10.2 mmol/L; ammonia 87 µmol/L; creatinine 1.48 mg/dL
4	Extubation Labs: creatinine 4.71 mg/dL
5	Consultation‐liaison psychiatry team consulted, started as needed haloperidol for severe agitation and scheduled melatonin 3 mg at bedtime for sleep–wake cycle
9	MRI brain: 1. Scattered small diffusion abnormalities in both cerebral hemispheres and bilateral cerebellar hemispheres with associated FLAIR hyperintensities, suggestive of scattered recent infarcts, which could be embolic in nature 2. Mild diffusion abnormality in the hippocampi, more so on the left, nonspecific, which could be postictal change, metabolic abnormality or hypoxic ischemic in nature
10	Ultrasound carotid duplex bilateral: no hemodynamically significant stenosis Labs: creatinine 5.1 mg/dL Home escitalopram 10 mg restarted for anxiety
16	MoCA: 12/30; primarily deficits in executive function, attention, and delayed recall Psychiatric sitter discontinued Labs: creatinine 2.19 mg/dL Trazodone 50 mg started at bedtime for insomnia
19	Scheduled dialysis held due to signs of renal recovery Labs: creatinine 1.33 mg/dL
22	PermCath removed due to renal recovery
25	Escitalopram and trazodone discontinued due to continued insomnia and racing thoughts Quetiapine 50 mg at bedtime started at bedtime
26	Quetiapine increased to 100 mg at bedtime
29	Quetiapine increased to 150 mg at bedtime Patient discharged home with partial hospitalization care starting the following week

Abbreviations: CRRT, continuous renal replacement therapy; CT, computed tomography; EEG, electroencephalogram; ICU, intensive care unit; MoCA, Montreal Cognitive Assessment; MRI, magnetic resonance imaging.

The ammonia level on admission was 548 µg/dL, which improved to 87 µg/dL following hemodialysis. Computed tomography of the abdomen and pelvis showed mild right hydronephrosis with mild fullness in the right ureter, which was possibly consistent with the passage of a urethral calculus. Urine drug screen, urine fentanyl screen, ethanol, and acetaminophen levels were negative. There was suspicion of toxic ingestion of ethylene glycol, given that lactate on arterial blood gas was significantly higher than serum lactate, and fomepizole was started. The ICU team reached out to the patient’s family due to concern for toxin ingestion, who reported that the patient had made suicidal statements prior to admission. The family subsequently found a suicide note alongside antifreeze (⅔ of the bottle missing) and rat poison (1–2 tablets missing) in the patient’s bedroom. Poison control recommended continuing fomepizole until the labs normalized.

Following extubation a few days later, the consultation‐liaison psychiatry service was consulted due to the suicide attempt. The patient was only oriented to self and daughter at that time and reported that she could not remember any events leading up to hospitalization, including the suicide attempt. She provided brief responses, had poor attention, and required multiple attempts and repetition of questions to receive responses, which was assumed to be due to delirium. Per chart review, the patient’s home medications included trazodone 150 mg at bedtime for insomnia and escitalopram 10 mg daily for depression. Prior psychotropic trials included aripiprazole 5 mg daily a year prior and quetiapine of unknown dose at some point for sleep. Due to impaired mental status, only melatonin 3 mg at bedtime was started for the sleep–wake cycle. Over the next few days, the patient’s cognition improved slowly, but she was consistently not able to retain memory of the year, being in the hospital, or medical conversations from the day prior. In addition, a family member noticed that the patient’s left eye would “look out” compared to her right eye and that she would “zone out” intermittently, which they reported was also happening in the ICU. The psychiatry service recommended brain magnetic resonance imaging (MRI) to rule out acute neurological pathology contributing to slow cognitive recovery. The MRI revealed multiple lacunar infarcts in bilateral hemispheres, appearing as scattered small mild diffusion abnormalities in both cerebral hemispheres, predominantly involving the periventricular, deep subcortical white matter, and scattered foci in the bilateral cerebellar hemispheres with associated fluid‐attenuated inversion recovery (FLAIR) hyperintensities, with mild diffusion abnormality in the hippocampi, more so on the left. The neurologist felt this was likely secondary to anoxic brain injury after the toxic ingestion of ethylene glycol and rat poison. The Montreal Cognitive Assessment (MoCA) score was 12/30, with most deficits in executive function, attention, and delayed recall. Due to continued hemodialysis with unclear renal recovery, poor cognitive status, inability to remember her suicide attempt, and no mood disturbances noted during psychiatric evaluation, inpatient psychiatric treatment became less reasonable. Trazodone 50 mg at bedtime was started for insomnia, and safe discharge planning was ongoing.

Over the next 2 weeks, the patient’s executive function improved, but she continued to struggle with declarative memory retrieval, attention span, and abstract thinking. Given the patient’s cognitive improvements, ability to retain new information, and ability to now reflect on past mental health struggles, the psychiatry service recommended a partial hospitalization program. The patient began to slowly recall her prior depressive thoughts and agreed to restart escitalopram 10 mg daily. Three weeks after admission, her renal function improved dramatically, and hemodialysis was discontinued. Around this time, escitalopram was discontinued due to concerns about activation and poor sleep, as well as new patient and family reports concerning a possible history of mania. Due to the increased likelihood of the patient having a bipolar spectrum disorder, escitalopram and trazodone were discontinued, and quetiapine 50 mg at bedtime was started and titrated to 100 mg within 2 days, with significant improvement in racing thoughts and insomnia. The patient was discharged to a partial hospitalization program on quetiapine 150 mg at bedtime after almost 4 weeks of hospitalization.

## 3. Discussion

The hospital course of this patient highlights a reported ingestion of two toxic substances, ethylene glycol and cholecalciferol. The patient in this case was found to have severely elevated anion‐gap metabolic acidosis, with suspicion for ethylene glycol poisoning due to discrepancies in lactate levels. The discovery of antifreeze and rat poison in the patient’s room alongside a suicide note highly suggested this etiology of the patient’s presentation. Psychiatric disposition was limited by the patient’s impaired cognition and need for hemodialysis. The patient’s cognition did not improve over the next few weeks as expected, and an MRI of the brain found signs of an anoxic brain injury. The patient’s psychotropic regimen was adjusted secondary to concern for mania and possible history of bipolar disorder, and as the patient’s mentation and renal function improved, the patient was recommended for participation in a psychiatric partial hospitalization program. This case demonstrates the outcomes of ingestion of both ethylene glycol and cholecalciferol with multiple aspects of medicine, including toxicology, neurology, and psychiatry.

### 3.1. Toxicology

Although there has been no literature on the combination of both rat poison and ethylene glycol to our knowledge, literature has been published on the ingestion of the two substances alone. On review of the literature for ingestion of ethylene glycol, the patient similarly exhibited acidosis with elevated lactate, altered mental status, and acute kidney injury, escalating to the need for hemodialysis [10, 13]. Those who are more often found to be intentionally exposed to ethylene glycol are women, and older adults typically experience major side effects of ingestion [10]. There are three postulated stages of ethylene glycol poisoning: the neurological stage, which transpires from a range of 30 min to 12 h after poisoning, the cardiopulmonary stage, developing from 12 to 24 h after poisoning, and the renal stage, occurring 24–72 h after the poisoning [15].

Regarding the ingestion of rat poison, the composition of d‐CON has changed over time. Per documentation in this case, the patient ingested rat poison consisting of d‐CON, with an active ingredient of cholecalciferol. There has been literature published on suicide attempts with d‐CON, but the predominant focus thus far has been on d‐CON, primarily consisting of brodifacoum, a vitamin K antagonist that was the active component of d‐CON prior to 2018 [3, 5–7, 9]. In a case report detailing the outcomes of accidental ingestion of cholecalciferol in a child, the child was asymptomatic with stable values even after follow‐up months later, although this was attributed to appropriate immediate treatment [16]. A case report of two dogs revealed hypercalcemia after ingestion of rat poison composed of cholecalciferol, whereupon one dog died from the poison and a second recovered after prolonged treatment [17]. Not all exposures to brodifacoum in the literature have led to significant bleeding or death [5]. A case report of a suicide attempt with intentional ingestion of 0.005% brodifacoum via consuming 168 g of d‐CON Mouse‐Prufe I1 caused hemorrhages in the pleura and pericardium, and a repeated exposure 15 weeks later to more of the brodifacoum in a subsequent suicide attempt caused a subarachnoid hemorrhage, resulting in the patient’s death [3]. Another case report of a child involved bleeding from the patient’s urinary tract, mouth, and nose secondary to erroneous brodifacoum exposure [7]. Our case study demonstrates that exposure to cholecalciferol via d‐CON was not nearly as problematic for our patient as was her exposure to ethylene glycol, and the purported impacts of cholecalciferol pale in comparison to those of brodifacoum. In 2018, the active ingredient in d‐CON was officially changed to cholecalciferol, also known as vitamin D3 [9]. From our literature review, there is no reported research on the usage of cholecalciferol as a means of a suicide attempt in the context of rat poison. A study by Rumbeiha et al. [18] postulated that both ethylene glycol poisoning and cholecalciferol could result in elevated serum calcium, while only cholecalciferol is associated with elevated serum phosphate. Thus, a combination of both cholecalciferol and ethylene glycol could result in both elevated calcium and phosphorus in the blood and kidney. Although the ratio of total renal calcium to total phosphorus could help distinguish cholecalciferol ingestion from ethylene glycol ingestion, in this case, the combination would make this distinction irrelevant.

There have been reports of vitamin D toxicity, which appears as hypercalcemia in humans [8]. Our patient presented with altered mental status, and her imaging results showed potential passage of a urethral calculus, which would have been consistent with vitamin D toxicity [8]. Although most vitamin D toxicity cases conclude without grave complications, it can cause acute renal failure, which did occur in our patient [8]. In a case report examining exposure to brodifacoum in a suicide attempt, the patient presented with bilateral renal and ureteral thrombus resulting in nephropathy, which would be similar to our patient’s presentation with anuria, even though our patient was exposed to ethylene glycol [6]. However, the patient on admission had hypocalcemia that down‐trended and later normalized to normal serum levels, which is inconsistent with reports of hypercalcemia in severe vitamin D toxicity. Hypocalcemia is associated with ethylene glycol poisoning, which likely counteracted any effects of vitamin D [8, 14]. Since there are no documented cases of ingestion of cholecalciferol via rat poison or vitamin D toxicity in the context of a suicide attempt, we can only compare this patient to the literature published on vitamin D toxicity. Vitamin D toxicity often presents with vague symptoms, including altered mentation, weakness, abdominal symptoms such as pain and vomiting, and renal dysfunction such as nephrolithiasis and polyuria [8]. Nonetheless, it is much more likely that the patient’s ethylene glycol was also a major contributor to this patient’s renal dysfunction rather than the exposure to cholecalciferol. Of note, the patient’s slower recovery could have been exacerbated by delayed medical care as her family’s discovery of altered mental status and slurred speech over the phone resulted in subsequent evaluation of the patient in person. Thus, it is unclear how much time passed between the suicide attempt and time of the treatment.

### 3.2. Neurology

As the patient’s hospitalization progressed, psychiatry and neurology services were both consulted at different times. Psychiatry was consulted for concern of a suicide attempt after the patient was extubated; however, the patient struggled to answer questions and needed significant prompting from the interviewer. While in the ICU, the patient began to move spontaneously, but this appeared without purpose; thus, the psychiatry team recommended a brain MRI if mentation did not improve as expected. Secondary to elevated creatinine, MRI was originally deferred to optimize timing with improved renal function (given the need for contrast); however, the patient’s family reported to the psychiatry team that one eye’s movement was not consistent with the patient’s baseline. This concern resulted in escalation of priority for MRI, with deferral of contrast to preserve renal function, which showed multiple diffusion abnormalities consistent with anoxic brain injury likely from hypotension in the ICU. Patients with ethylene glycol poisoning have presented with delayed cranial nerve deficits per previous literature, consistent with our patient’s difficulty with eye movement in one eye [11, 15, 19]. As discussed previously, the neurological stage of ethylene glycol poisoning involves unmetabolized ethylene glycol leading to inebriation, cerebral edema, and calcium oxalate crystal accumulation in blood vessels [15]. Imaging studies have found involvement of the hippocampus during completion of activities dependent on episodic memory [20]. Hippocampus damage would thus be associated with episodic memory deficits, which is consistent with the patient’s lack of memory of the suicide attempt and slow cognitive recovery [20].

### 3.3. Psychiatry

The psychiatric care of this patient was complicated by two major issues: her prolonged hospitalization with significant cognitive deficits and her need for hemodialysis. The patient presented after a significant suicide attempt, which would typically result in a recommendation for inpatient psychiatric hospitalization. Although the patient was willing to go to an inpatient psychiatric unit, she continuously forgot about this conversation with the psychiatry team, and her cognitive deficits would have made intensive psychotherapy challenging. When medically cleared for discharge, the patient reported no recall of the suicide attempt or the note she had written.

The patient’s need for hemodialysis created a barrier to admission to local psychiatric units. There were also financial barriers due to the patient’s insurance status, preventing acceptance into psychiatric units able to accommodate hemodialysis. Following improvement in cognition and renal function, the utility of voluntary psychiatric hospitalization was questionable, considering the patient’s psychiatric stability during her prolonged hospitalization. With improved cognition, the decision was made to refer the patient to a partial hospitalization program for additional psychiatric care in a less‐restricted setting. This case illustrates the significant difficulties of finding appropriate acute psychiatric treatment for a patient with a significant medical illness, especially with a scarcity of med‐psych units. The struggle to secure psychiatric disposition for patients with medical needs, such as dialysis, requiring psychiatric hospitalization has been documented in news articles [21]. The cost of providing medical care to psychiatric patients is cited as a reason for exclusion of patients with medical comorbidities from psychiatric units, and proposed solutions include stratification of psychiatric hospitals by expertise level, similar to what has been done in trauma hospitals, and providing additional funding for psychiatric units [21].

Although the patient had been previously diagnosed with bipolar disorder, there were no records of this, and it was difficult to fully establish a primary mood disorder due to the patient’s history of substantial alcohol use, even up until the suicide attempt. The patient was restarted on her home escitalopram due to reporting benefits in the past; however, toward the end of her hospitalization, the patient complained of poor sleep and anxiety. The patient had previously taken trazodone, which was also restarted during the hospitalization. These medications were discontinued when the psychiatry team noted euphoria with rapid speech, concerning for the beginning of a manic episode. With improved cognition and ability to gain more clarity on prior symptoms, the patient reported a history of times with less need for sleep, impulsive and risky behavior even during long periods of alcohol sobriety, and a positive family history for bipolar disorder. She reported previously being treated with aripiprazole and quetiapine, with quetiapine having improved her sleep, but she stopped herself due to improvements in mood and insomnia. The patient’s escitalopram and trazodone were discontinued, and she was started on quetiapine for presumed bipolar disorder. Of note, there is also a relationship between the development of mania and anoxic brain injury. A systematic review conducted to review the onset of mania after a traumatic brain injury revealed 74% of patients within 1 year acquired mania, highlighting that the mania that the patient discussed in this case report experienced was likely secondary to the brain injury she acquired through her suicide attempt [22, 23]. Ultimately, the patient’s prolonged hospitalization allowed for diagnostic clarification.

## 4. Conclusion

Toxicologic, neurologic, and psychiatric factors contributed to the complex course of assessment and treatment after the presented patient’s suicide attempt. This case serves as an example of treatment modalities and psychosocial considerations of care that may serve to help guide care for similar patients in the future.

## 5. Patient Perspective

“I have to be honest that I don’t remember anything that led to me wanting to take my life…but I’m doing much better.”

“I’ve been using a cane and will start physical therapy soon.”

“I don’t recognize the faces of some people anymore and can’t drive due to not being able to remember addresses…[but] I am just really thankful for the staff that didn’t give up on me.”

The patient also shared that she is going to the recommended partial care psychiatric program for therapy to improve her memory. Since her suicide attempt, she denies cravings for alcohol. She continues to be on the same medication regimen and reports doing well overall.

## Author Contributions

**Danah Atassi**: conceptualization, methodology, project administration, software, writing – original draft, review and editing. **Amanda Bernardini and Wesley Wang**: writing – review and editing. **Christie Richardson**: writing – review and editing, supervision.

## Funding

No funding was received for this manuscript.

## Disclosure

The preliminary data of this manuscript were presented as a poster at Inspira Medical Center Research Day 2025 prior to publication.

## Ethics Statement

This case report was approved as an exempt determination by the Institutional Review Board at Inspira Health (IRB File Number 2025‐05‐008). Written informed consent was obtained from the patient for the publication of this case report. Permission was granted by Montreal Cognitive Assessment Cognition to use this score in this research project.

## Conflicts of Interest

The authors declare no conflicts of interest.

## Supporting Information

Additional supporting information can be found online in the Supporting Information section.

## Supporting information


**Supporting Information** The CARE checklist is included as supporting information available online.

## Data Availability

The data that support the findings of this study are available from the corresponding author upon reasonable request.
