# Supplementary material for: Suicide Attempt by Dual Ethylene Glycol and Rat Poison Ingestion: Case Report
Source: Case Rep Psychiatry. 2026 Jul 30;2026:5846563. doi: 10.1155/crps/5846563 (PMC13420282; doi:10.1155/crps/5846563)
Supplement: Supplementary file 1 — Supporting Information The CARE checklist is included as supporting information available online. [file CRPS-2026-5846563-s001.pdf]

CARE Checklist of information to include when writing a case report

CARE case report guidelines

(cc) BY-NC-ND

Topic	Item	Checklist item description	Reported on Line
Title	1	The diagnosis or intervention of primary focus followed by the words "case report"	1
Key Words	2	2 to 5 key words that identify diagnoses or interventions in this case report, including "case report"	31
Abstract (no references)	3a	Introduction: What is unique about this case and what does it add to the scientific literature?	35 - 36
	3b	Main symptoms and/or important clinical findings	36 - 41
	3c	The main diagnoses, therapeutic interventions, and outcomes	41 - 48
	3d	Conclusion—What is the main "take-away" lesson(s) from this case?	48 - 56
Introduction	4	One or two paragraphs summarizing why this case is unique (**may include references**)	60 - 87
Patient Information	5a	De-identified patient specific information.	91 - 95
	5b	Primary concerns and symptoms of the patient.	93 - 97
	5c	Medical, family, and psycho-social history including relevant genetic information	91 - 92, 116 - 119, 145 - 146
	5d	Relevant past interventions with outcomes	117 - 119
Clinical Findings	6	Describe significant physical examination (PE) and important clinical findings.	95 - 110, 119 - 133
Timeline	7	Historical and current information from this episode of care organized as a timeline	91 - 150
Diagnostic Assessment	8a	Diagnostic testing (such as PE, laboratory testing, imaging, surveys).	93 - 97, 101 - 105, 125 - 131
	8b	Diagnostic challenges (such as access to testing, financial, or cultural)	120 - 125, 144 - 146
	8c	Diagnosis (including other diagnoses considered)	105 - 110, 131 - 132, 146
	8d	Prognosis (such as staging in oncology) where applicable	149 - 150
Therapeutic Intervention	9a	Types of therapeutic intervention (such as pharmacologic, surgical, preventive, self-care)	97 - 99, 106, 119 - 120, 136, 143 - 149
	9b	Administration of therapeutic intervention (such as dosage, strength, duration)	19 - 120, 136, 143 - 149
	9c	Changes in therapeutic intervention (with rationale)	144 - 149
Follow-up and Outcomes	10a	Clinician and patient-assessed outcomes (if available)	N/A
	10b	Important follow-up diagnostic and other test results	149 - 150
	10c	Intervention adherence and tolerability (How was this assessed?)	143 - 149
	10d	Adverse and unanticipated events	120 - 132, 143 - 149
Discussion	11a	A scientific discussion of the strengths AND limitations associated with this case report	155 - 167
	11b	Discussion of the relevant medical literature **with references**.	169 - 286
	11c	The scientific rationale for any conclusions (including assessment of possible causes)	169 - 286
	11d	The primary "take-away" lessons of this case report (without references) in a one paragraph conclusion	288 - 291
Patient Perspective	12	The patient should share their perspective in one to two paragraphs on the treatment(s) they received	~~N/A~~ 285-292
Informed Consent	13	Did the patient give informed consent? Please provide if requested	Yes ☒ No ☐
